# Influence of Cellulose Particles on *Streptomyces* spp. Growth, Morphology, and Metabolite Spectrum

**DOI:** 10.1111/1751-7915.70445

**Published:** 2026-09-16

**Authors:** Dorothea M. Schütterle, Bashar Al‐Smadi, Jethro L. Hemmann, Gesa Brauneck, Ana M. Palacio‐Barrera, Ivan Schlembach, Sascha Schäuble, Jorgen Barsett Magnus, Gianni Panagiotou, Miriam A. Rosenbaum

**Affiliations:** ^1^ Bio Pilot Plant Leibniz Institute for Natural Product Research and Infection Biology, Hans‐Knöll‐Institute Jena Germany; ^2^ Faculty of Biological Sciences Friedrich‐Schiller‐University Jena Jena Germany; ^3^ Department of Microbiome Dynamics Leibniz Institute for Natural Product Research and Infection Biology, Hans‐Knöll‐Institute Jena Germany; ^4^ Metabolomics‐Guided Natural Product Discovery Leibniz Institute for Natural Product Research and Infection Biology, Hans‐Knöll‐Institute Jena Germany; ^5^ AVT – Biochemical Engineering RWTH Aachen University Aachen Germany; ^6^ Cluster of Excellence Balance of the Microverse Friedrich‐Schiller‐University Jena Jena Germany

**Keywords:** cellulose, metabolomics, morphology, *Streptomyces*

## Abstract

Filamentous soil bacteria of the genus *Streptomyces* are a major source of bioactive natural products, including antibiotics. However, their industrial cultivation is complicated by complex morphologies ranging from dispersed mycelia to dense pellets, which are closely linked to productivity. Microparticles, such as talc or aluminium oxide, are commonly added to modulate macromorphology, yet their effects across diverse *Streptomyces* species remain insufficiently characterized. Here, we systematically evaluated the impact of cellulose particles on growth, morphology, and metabolite production in 13 *Streptomyces* species (*Streptomyces* spp.). Cellulose addition significantly reduced pellet size and altered metabolite profiles in a species‐dependent manner, resulting predominantly in a decrease of natural product abundance. Without cellulose addition, metabolite profiles correlated with biosynthetic potential encoded in the genomes, whereas this relationship did not hold in the presence of cellulose. With the addition of cellulose, a decrease in peptide signals present across species was observed. Cellulose‐associated mechanical stress led to species‐specific metabolomic shifts characterized by increased amino acids, lipids, and other degradation products, consistent with enhanced mechanical cellular disruption, which might have decreased proteolytic turnover during the stationary phase in some species that were more prone to mechanical stress. Although pellet size reduction is often associated with increased abundance of natural products, these findings demonstrate that the influence of solid particles on *Streptomyces* natural products and metabolism is species‐dependent. This work provides a foundation for future studies using dynamic systems, such as co‐cultivation with cellulolytic organisms, to improve natural product discovery.

## Graphical abstract

Cellulose particles reduce pellet size and alter morphology in 13 *Streptomyces* spp., but induce species‐dependent metabolic responses that frequently decrease natural product production. Cellulose‐associated mechanical stress disrupts the relationship between biosynthetic potential and metabolite output, leading to stress‐associated metabolomic shifts characterized by increased amino acids, lipids, and degradation products.

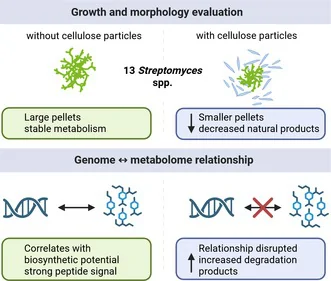

## 1 Introduction

Over the course of the last century, a plethora of medically and biotechnologically useful compounds have been derived from natural products originally discovered in microorganisms. A very prominent source of natural products are Actinomycetes, whereby about 80% of actinomycete‐derived antibiotic natural products originate from the genus of *Streptomyces* (Alam et al. 2022; Kieser et al. 2000). Although antibiotic discovery rates in *Streptomyces* have declined in recent decades (Alkatheri et al. 2023), ongoing methodological advances for screenings and induction can still result in the discovery of novel antibiotics (Antoraz et al. 2015; Watve et al. 2001). Secondary metabolite production in *Streptomyces* is tightly regulated, often by the availability of environmental nutrients such as carbon, nitrogen, and phosphate. High concentrations of carbon sources (e.g., glucose) often lead to catabolic repression of secondary metabolites. While phosphate and nitrogen limitations trigger morphological differentiation, such as the formation of aerial mycelia in cultures grown on solid media, which leads to cell cycle progression and secondary metabolite production (Romero‐Rodriguez et al. 2018).

The *Streptomyces* life cycle resembles that of filamentous fungi, with spore germination, elongation into germ tubes, and differentiation into branched hyphal networks (Bobek et al. 2017). For most filamentous organisms, growth morphologies span from dispersed growth to clump‐like morphologies to compact pellets of varying sizes (Bol et al. 2021). It is widely debated which macromorphology is particularly advantageous for secondary metabolite production, and this further varies between *Streptomyces* spp. A pelleted morphology, for example, increased nikkomycin production in *Streptomyces tendae* and avermectin production in *Streptomyces avermitilis* (Vecht‐Lifshitz et al. 1992; Yin et al. 2008). In contrast, a more dispersed morphology led to increased geldanamycin and tylosin production for *Streptomyces hygroscopicus* and *Streptomyces fradiae* , respectively (Dobson et al. 2008; Tamura et al. 1997). In fermentations with varying stirrer speeds, morphological changes due to increased hydromechanical stress did not significantly affect product titers (Belmar‐Beiny and Thomas 1991; Yang et al. 1996). Hydrodynamic shear in stirred tank fermenters is mainly generated in localized high‐energy dissipation zones around the impeller, where hyphal breakage can occur if the smallest turbulent eddies approach the dimensions of the hyphae and exceed their mechanical strength (Schrader et al. 2023). Because hyphal strength depends on the physiological state and morphology of the mycelium, no universal shear threshold for breakage has been established (Li et al. 2002). In contrast, shaken cultivation systems exhibit a more homogeneous distribution of power dissipation and substantially lower local energy dissipation rates than stirred‐tank bioreactors, generally resulting in lower hydrodynamic stresses and larger pellet sizes (Schrader et al. 2023). A scale‐up study of various *Streptomyces* spp. showed that keeping the hydromechanical stress constant can improve morphological consistency across scales (Brauneck et al. 2026). These results highlight the variety of responses between *Streptomyces* spp. to cultivation conditions and shear stress. Morphology engineering aims to improve productivity by achieving specific morphological growth forms. One strategy to modulate macromorphology in filamentous organisms is the addition of solid particles in microparticle‐enhanced cultivations. Depending on their size, these particles are commonly classified as microparticles (< 50 μm) or macroparticles (> 50 μm), which influence morphology through different mechanisms (Bol et al. 2021). Even though filamentous bacteria are underrepresented in this type of cultivation, for *Streptomyces aureofaciens* it was possible to reduce the pellet size by adding aluminium oxide (Al_2_O_3_) microparticles (Kaup et al. 2008). *Streptomyces* M‐Z18 produced 50% more ɛ‐poly‐L‐lysine with the addition of talc in shake flask experiments (Ren et al. 2015). Modelling of macroparticles, such as glass beads, in shake flasks revealed that mechanical stresses arose through repeated particle–hypha and particle–particle collisions, with the highest shear stresses occurring at bead‐bottom contact surfaces (Schrader et al. 2023). The addition of glass beads reduced pellet diameter and increased geldanamycin concentration in cultivations of *S. hygroscopicus* var. *geldanus* (Dobson et al. 2008). An increase in undecylprodigiosin and actinorhodin was observed in *Streptomyces coelicolor* with the addition of 3 mm glass beads, resulting in dispersed growth (Sohoni et al. 2012). Rather than a universal productivity threshold, the effect of macroparticle‐induced mechanical stress on secondary metabolite production appears to follow an optimum that depends on the producing organism, the target metabolite, and the physiological state of the biomass (Schrader et al. 2023). This effect is often attributed to increased nutrient and oxygen supply to dispersed mycelia (macroparticles) or to a loosened pellet core (microparticles), thereby increasing active mycelium and, consequently, productivity.

To better understand cultivation processes and growth dynamics, online monitoring of microbial cultivations became a standard technique. Optical methods, including optical density, scattered‐light, and fluorescence, are used because they are noninvasive and easy to implement online. While scattered‐light is nearly linearly correlated with cell concentration and is suitable for biomass measurements for most microorganisms (Geinitz et al. 2020), the composition of the cultivation medium, such as turbidity and solid particles, can adversely affect the accuracy of scattered‐light measurements (Schlembach et al. 2021). As a solution, autofluorescent metabolic compounds, such as flavins, can be employed to track biomass formation (Bogomolov et al. 2011; Masiero et al. 2012). Brauneck et al. showed that *S. coeruleorubidus* and *S. bobili* produce autofluorescent molecules enabling online growth monitoring and discrimination of co‐culture partners during cultivation (Brauneck et al. 2025).

**FIGURE 1:**
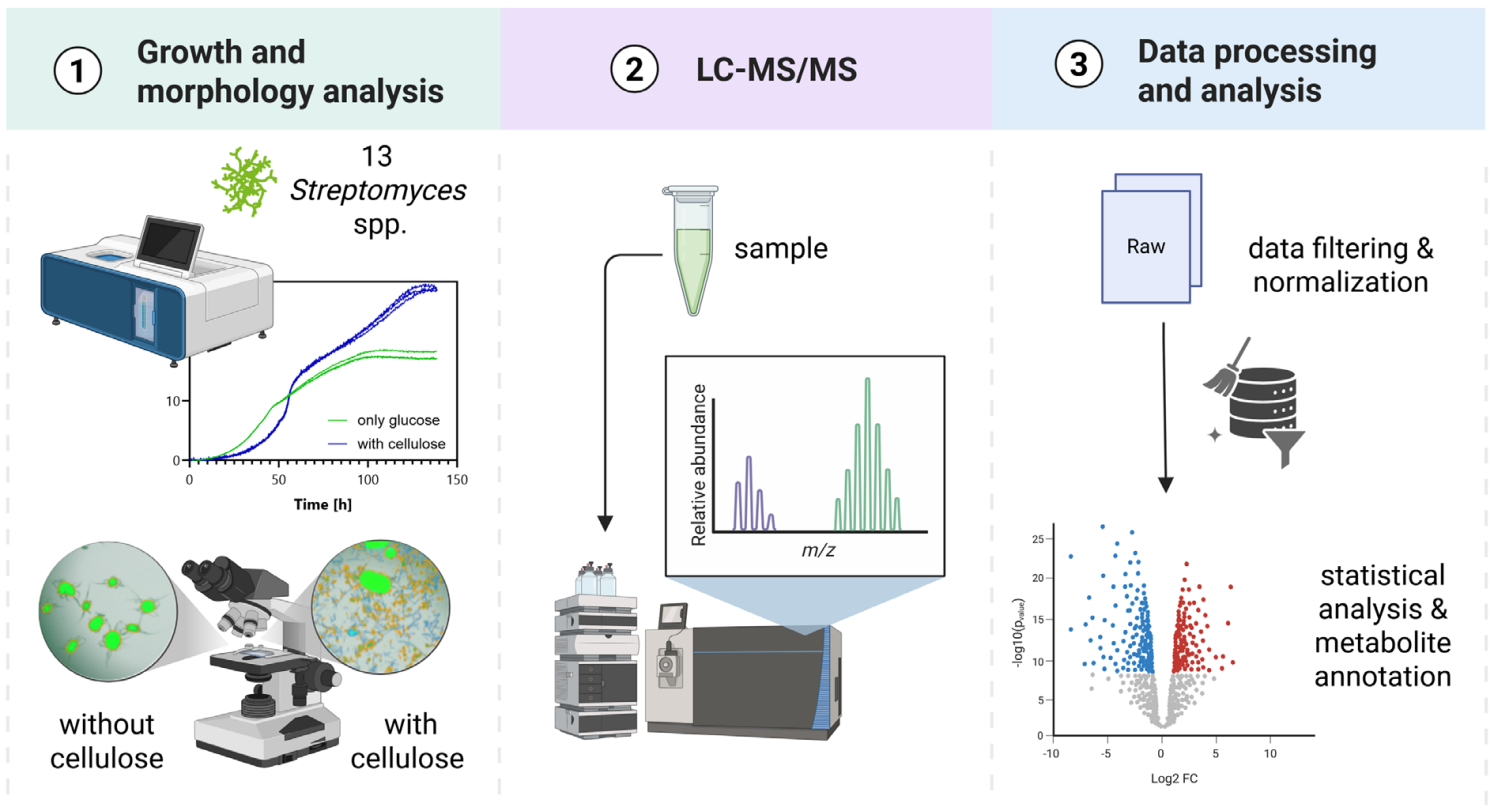
Experimental approach to study the influence of cellulose particles on *Streptomyces* spp. (1) 13 different *Streptomyces* spp. were cultivated on glucose in the presence and absence of cellulose. The effect of cellulose on macromorphology and growth behaviour was quantified. (2) Samples were extracted and analysed via liquid chromatography—mass spectrometry (LC–MS). (3) After data normalization and filtering, statistical modelling and chemical class annotation were performed.

While previous studies utilizing microparticles have been shown to modulate secondary metabolite production in individual *Streptomyces* spp., these investigations are restricted to a small number of model organisms and targeted metabolites. While the metabolic responses seem to differ between species, it is currently unclear if there is a shared mechanism that can be investigated using a more systematic approach, such as untargeted metabolomics. In contrast to previously studied inorganic particles such as Al_2_O_3_, talc, or glass beads, cellulose fibres differ substantially in their size, density, surface properties, and biodegradability; it is therefore unclear whether they influence morphology and secondary metabolism through comparable mechanisms. Here, we explored the growth parameters and morphological phenotype of 13 *Streptomyces* spp. and investigated the effect of cellulose as a solid particle on these species and their metabolome (Figure 1), representing, to our knowledge, the first application of cellulose to alter morphology in filamentous organisms. Genome‐mining of biosynthetic potential across all species will be performed to assess whether metabolic patterns correlate with phylogeny.

## 2 Material and Methods

### 2.1 Microorganisms

All *Streptomyces* spp. were obtained from the Leibniz‐HKI culture collection (Jena, Germany). The *Streptomyces* spp. used were: *Streptomyces avermitilis* DSM 46492, *Streptomyces fradiae* DSM 40063, *Streptomyces rimosus* DSM 40260, *Streptomyces alboniger* DSM 40043, *Streptomyces coeruleorubidus* DSM 40145, *Streptomyces filamentosus* DSM 40022, *Streptomyces collinus* DSM 40733, *Streptomyces nodosus* DSM 40109, *Streptomyces cinereoruber* ST010814, *Streptomyces venezuelae* DSM 40230, *Streptomyces nitrosporeus* DSM 40023, *Streptomyces bobili* DSM 40481 (heterotypic synonym *Streptomyces galilaeus* (Saygin et al. 2020)), and *Streptomyces albofaciens* DSM 40268.

### 2.2 Medium Composition

The medium used in this study is low nitrogen phosphate (LNP) medium, which has been described previously (Finger et al. 2022). It contains the following components: (NH_4_)_2_SO_4_ 3 g L^−1^, urea 0.3 g L^−1^, KH_2_PO_4_ 0.4 g L^−1^, MgSO_4_·7H_2_O 0.5 g L^−1^, CaCl_2_·2H_2_O 0.23 g L^−1^, NaCl 0.05 g L^−1^, MES buffer 0.1 M, Tween 80 0.01% (v/v), trace element solution 2.5 mL L^−1^. The pH was adjusted with NaOH to 6.7. The trace element solution was comprised of citric acid 180 g L^−1^, ZnSO_4_·7H_2_O 16 g L^−1^, CoCl_2_·6H_2_O 2.71 g L^−1^, Fe_2_(SO_4_)_3_ 2.29 g L^−1^, CuSO_4_ 2.05 g L^−1^, MnSO_4_·7H_2_O 1.6 g L^−1^, H_3_BO_3_ 0.8 g L^−1^. The medium contained 30 g L^−1^ glucose for all cultivations and was sterile‐filtered through a 0.2 μm pore‐size PES membrane filter (Labsolute, Germany).

### 2.3 Cultivations in Microtiter Plates

Growth experiments were conducted on the BioLector I device (Beckman Coulter). All cultivations were performed in 48 round‐well microtiter plates (M2P‐MTP‐R48‐B) and sealed with gas‐permeable membranes (M2P‐F‐GPR48‐10). The *Streptomyces* spp. were initially cultivated in 50 mL straight rim flasks in 10 mL M79 complex medium (Prauser and Falta 1968) at 140 rpm for 7 days. For pre‐cultures, 10% (v/v) of this culture was inoculated into 50 mL straight rim flasks with 5 mL LNP (30 g L^−1^ glucose) and 15 sterile stainless‐steel beads (2–2.5 mm) to disperse the biomass. The cultures were incubated at 28°C at 140 rpm for 48 h (shaking diameter of 50 mm). The biomass was collected by centrifugation in 15 mL tubes, washed twice with distilled water, and then transferred to 2 mL screw‐cap tubes (Heathrow Scientific, USA) that contained 250 mg of pre‐sterilized 0.5 mm glass beads. To ensure a uniform and dispersed inoculum, the biomass was homogenized at 4 m s^−1^ for 3 s using a FastPrep (Precellys 24, Bertin Technologies, France). For each species, the optical density (OD) was measured at 600 nm (BioPhotometer, Eppendorf, Germany) and adjusted to 2. From this stock solution, microtiter plate cultivations in LNP (30 g L^−1^ glucose) were inoculated to an initial OD of 0.1. To evaluate the influence of cellulose as a solid particle on growth behaviour and macromorphology, three biological replicates per species were used for each condition (with and without addition of 30 g L^−1^ α‐cellulose (Sigma‐Aldrich, Germany)). The α‐cellulose was pre‐weighted into 1.5 mL Eppendorf tubes, sterilized, and added to each well under sterile conditions. The cultivation conditions were as follows: 800 rpm, 3 mm shaking diameter, 30°C, duration up to 150 h. During cultivations, optical measurements were taken in different spectral channels, such as scattered‐light (Ex: 620 nm, Em: 620 nm) as an indicator of biomass and HPTSII (Ex: 450 nm, Em: 520 nm) as an indicator of flavin autofluorescence. The gain settings are shown in Table S1.

### 2.4 Growth Rate Calculations

To determine whether autofluorescence can be used as a measure of biomass rather than scattered‐light measurements, which are unsuitable when solid particles are present, we compared the initial growth rate *μ*_1_ derived from both scattered‐light and autofluorescence of the cultivations. For this purpose, cultivations without cellulose particles were blanked against the median of the non‐inoculated glucose medium measurements. The cellulose particle cultivations were blanked by subtracting an initial median starting value (of the time range 2.5–5.02 h) of each well, because the cellulose concentration differed slightly in each well due to weighing error. MATLAB was used to process all growth curves. The autofluorescence curve of each replicate was divided into growth segments using the *findchangepts* function, the slope was calculated by fitting a linear regression to each segment, and the same segments were analysed for the scattered‐light measurement. For the cellulose cultivations, only the autofluorescence curves were analysed because the scattered‐light noise level was too high during the initial measurement phase, attributable to the cellulose fibres. To compare initial growth rates *μ*_1_, *t*‐tests between the autofluorescence and scattered‐light (paired *t*‐test) and between the two conditions (without cellulose and with cellulose particles) were performed. As an indicator of the growth curve trajectory, the area under the curve (AUC) of the autofluorescence signal was compared between the two conditions.

### 2.5 Microscopy and Image Analysis

Microscopic images were obtained on a Keyence BZ‐X810 fluorescence microscope (Keyence, Japan). Technical duplicates were performed for each biological replicate. Cultivation broth was diluted with deionized water (1:50 for cultures without cellulose and 1:100 for cultures with cellulose) in a 96‐well plate (Greiner) using wide‐bore pipette tips to ensure sufficient transfer of pellet biomass. To stain the cellulose fibres, 10 μL of a 10 mg mL^−1^ calcofluorwhite solution (Fluorescent Brightener 28, MP Biomedicals) in 5 mg mL^−1^ KOH was added to each well. A Plan Apochromat 2× objective (Keyence, Japan) was used to capture the entire well; imaging conditions are shown in Table S2. Image analysis was performed with JIPipe (Gerst et al. 2023). For each *Streptomyces* spp., Feret diameter was analysed for all pellets of 6 replicate wells. Mann–Whitney tests were conducted to ascertain statistical differences in pellet Feret diameter between conditions. Statistical tests were performed using GraphPad Prism version 10.4.1 for Windows (GraphPad Software, Boston, Massachusetts, USA).

### 2.6 Metabolite Measurement by LC–MS

After storage at −20°C, samples were thawed and 400 μL of culture broth was transferred to a 2 mL tube, before 400 μL of 100% methanol (VWR chemicals, USA) was added. After vortexing, the samples were centrifuged for 10 min at 15,000 rpm (Microstar 17, VWR, USA). The supernatants were transferred to LC glass vials and stored at −20°C until LC–MS/MS analysis.

Metabolite measurements were performed on an UHPLC system (UltiMate 3000, Thermo Scientific) coupled with an Orbitrap mass spectrometer (Q Exactive Plus, Thermo Scientific). A reversed‐phase column (Phenomenex Luna Omega C18, 1.6 μm, 100 × 2.1 mm, with a SecurityGuard pre‐column) was used to chromatographically separate metabolites at 40°C. Mobile phase A was water with 0.1% formic acid, and mobile phase B was acetonitrile with 0.1% formic acid. The flow rate was 0.3 mL min^−1^ and the following gradient was applied: 0–1.5 min: 3% B; 1.5–16.5 min: 3% to 97% B; 16.5–20 min: 97% B; 20–20.5 min: decrease to 3% B; 20.5–24 min: 3% B. The injected sample volume was 5 μL. Mass spectrometric analysis was performed in positive mode, with heated electrospray ionization parameters set to: spray voltage 3 kV, sheath gas 35, aux gas 10, probe heater 300°C, capillary temperature 320°C, S‐lens RF level 50. For high resolution MS/MS, a full scan (150–1500 m/z, 70k resolution, AGC target 3e6, maximum injection time 100 ms) was recorded, followed by 10 data‐dependent TopN MS/MS scans (17.5k resolution, AGC target 1e5, maximum injection time 50 ms, isolation window 1.5 m/z, stepped normalized collision energy: 30, 40, minimum AGC target 3e3, charge exclusion: unassigned, 3–8, > 8, exclude isotopes: on, dynamic exclusion 10 s). To achieve a greater fragmentation depth, an exclusion list from medium controls was generated and applied. All data were recorded as centroids and converted into mzML format using MSConvert (Chambers et al. 2012). Feature finding was done using MZmine 4.7.29 (Schmid et al. 2023) with a batch file created using the processing wizard and adding an additional retention time correction step. Targeted metabolite analysis was performed for metabolites with sufficient annotation quality in SIRIUS (Table S5) (Dührkop et al. 2019). *t*‐Tests of peak areas were conducted between cultivation conditions.

### 2.7 Data Filtering and Analysis

For the untargeted metabolomics analysis, first, a statistical limit of blank inspired robust filter was applied to the raw peak areas (defined per‐feature as the median blank peak area plus 3 × median absolute deviation (MAD) across the methanol blank samples). Then, the samples were normalized using Probabilistic Quotient Normalization (PQN) (Dieterle et al. 2006; Pakkir Shah et al. 2025). Given that medium‐derived components dominated the LC–MS chromatograms, the reference spectrum was constructed from the median of total ion chromatogram (TIC)‐normalized peak areas of all samples and the 12 medium controls (excluding methanol blanks). To anchor the normalization to the technical baseline, PQN factors were rescaled so that the median factor of the medium controls equaled 1.0. Medium‐derived background features were then excluded using a sample‐to‐medium (S/M) ratio (based on the 95th‐percentile peak area) threshold of ≥ 10. To remove low‐quality features while explicitly protecting species‐specific signals, a two‐stage filtering process was implemented: a presence filter retained features detected above their blank limit in at least two replicates within any single experimental group, and a data‐driven filter based on the maximum of the median PQN‐normalized peak areas (threshold: 13,220) was implemented to remove low‐abundance noise while preserving species‐specific features. This same threshold was then applied as a noise flattening (censoring) step to neutralize condition‐dependent background differences (e.g., slightly higher background signals in cellulose‐containing samples). All intensities below it were set to that value exactly, ensuring that only signals exceeding the noise value contribute to the statistical model. After filtering, 50,783 features remained for subsequent statistical analysis.

A statistical analysis was conducted using the limma package in R (Ritchie et al. 2015). A linear model with empirical Bayes moderation (Benjamini‐Hochberg FDR, *α* = 0.05, and |Log2 Fold Change| ≥ 0.5) was applied to identify features that were significantly differentially abundant between the two cultivation conditions. The model included terms for cultivation condition and species identity to identify the effect of cellulose while statistically accounting for species‐specific differences in metabolite abundance. Quality control measures are described in Supporting Information, and results are shown in Figure S1. PCA was performed after feature filtering, log2 transformation, centering, and scaling.

To obtain biological insights into the metabolic profiles of all 13 *Streptomyces* spp., we implemented a SIRIUS 6.3.0 and CANOPUS pipeline (Dührkop et al. 2019, 2021). A critical refinement in this final analysis was the implementation of a multi‐stage annotation strategy to prevent the aggregation of distinct biological signals into generic ontology terms. Specifically, chemical classes were assigned by prioritizing the most granular ontology level available (e.g., ClassyFire Level 5, Subclass, or NPClassifier) over broader parent classes; related subclasses were then combined into custom broader categories used for biological interpretation in the figures and text. A high‐confidence annotation threshold for structure or class was defined as a SIRIUS CSI:FingerID confidence score (Hoffmann et al. 2022) or CANOPUS class posterior probability of ≥ 0.70, respectively, approximating Metabolomics Standards Initiative (MSI) Level 2 or 3 annotation (Schymanski et al. 2014). High‐confidence annotations are indicated by bold text in the visualizations.

### 2.8 Comparison of Biosynthetic Potential and Metabolomic Profile

A phylogenetic tree was created for reference genomes (NCBI accession numbers in Table S3) using the online tools autoMLST (Alanjary et al. 2019) and iTOL (Letunic and Bork 2024). Analysis of biosynthetic gene clusters was conducted by mining all *Streptomyces* genomes with antiSMASH 8.0.4 using a “relaxed” HMM detection stringency (Blin et al. 2025). Then BiG‐SCAPE 2.0.0 was used to cluster the biosynthetic gene clusters (BGCs) into Gene Cluster Families (GCFs; alignment mode: glocal; similarity cutoff: 0.3) (Draisma et al. 2026). Genomic distance was calculated using Jaccard dissimilarity (excluding singleton GCFs) and validated with a count‐based Bray–Curtis dissimilarity. For each species, replicate peak areas were averaged per condition, and metabolomic distance was calculated as the Euclidean distance between log2‐transformed PQN‐normalized peak areas from the condition without cellulose. The correlation between genomic and metabolic topologies was quantified using the Mantel test (Spearman rank correlation, 9999 permutations) and confirmed by procrustes analysis. Robustness was verified using a “Leave‐One‐Out” sensitivity analysis. To test whether the cellulose‐induced metabolic response correlated with biosynthetic potential, a response distance matrix was calculated (Euclidean distance on log2 fold changes between conditions) and compared to the genomic distance using three feature sets: all features, only significantly changed features (FDR < 0.05 and |log2 fold change| ≥ 0.5), and the top 500 features with the highest variance in their response across species.

## 3 Results and Discussion

### 3.1 Cellulose Decreases Pellet Size in Pellet‐Forming *Streptomyces* spp.

**FIGURE 2:**
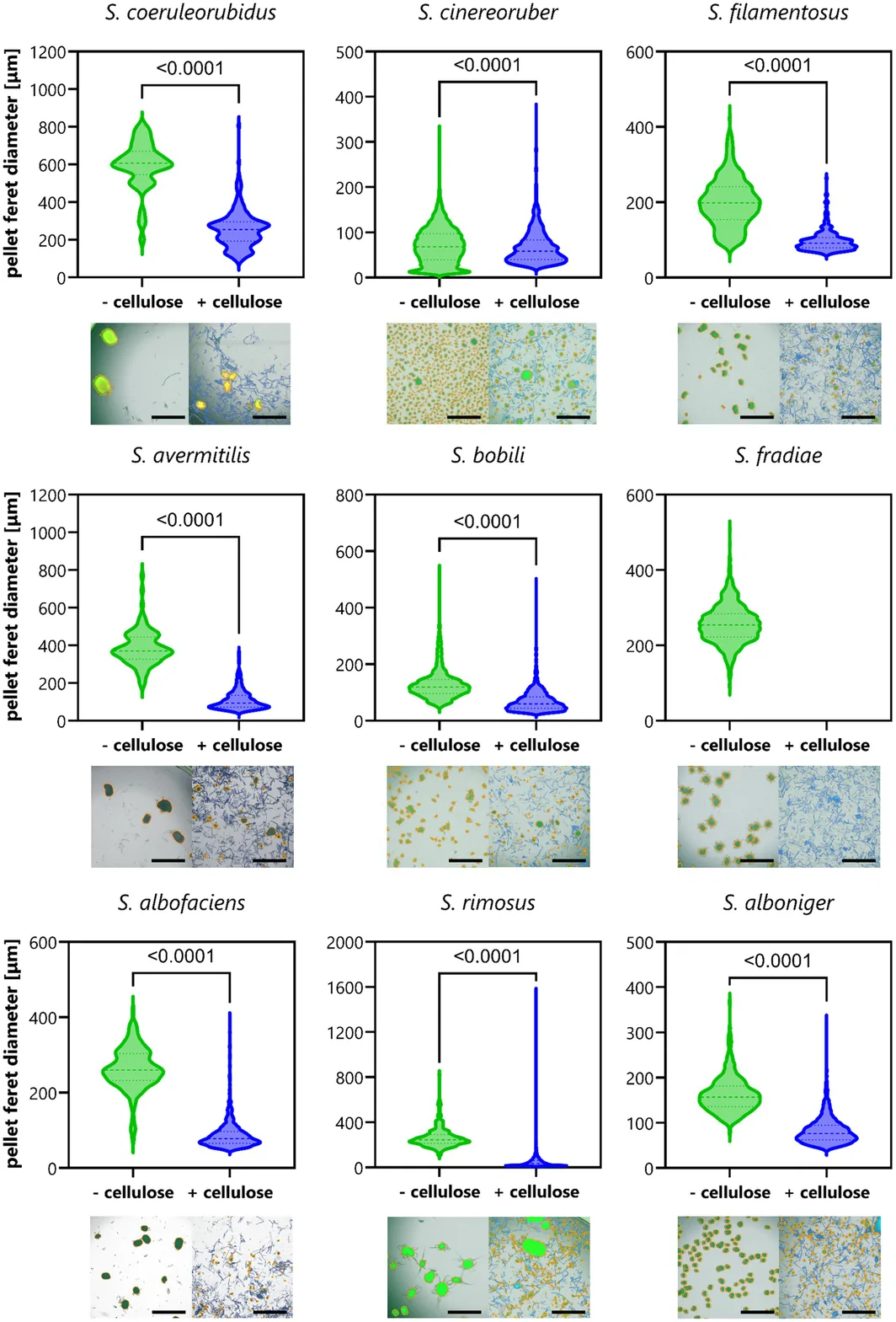
Effect of cellulose addition on macromorphology of pellet‐forming *Streptomyces* spp. grown on 30 g L^−1^ glucose. Statistical comparison (Mann–Whitney tests) of mean pellet Feret diameter with exemplary microscopy images (analysed particles indicated in orange, scale bars represent 1000 μm). Biological triplicates per species and condition. Image analysis was performed on two images per biological replicate.

We first investigated the growth morphology with and without cellulose fibres of 13 selected *Streptomyces* spp., most of which are known for the production of industrially relevant bioactive compounds (Lee et al. 2020). While genes related to plant biomass degradation are widespread in *Streptomyces*, the ability to rapidly deconstruct cellulose is rare outside of host‐associated *Streptomyces* species (Book et al. 2016). All species utilized in this study are free‐living soil isolates, with no indication of cellulolytic activity. In microtiter plates on nutrient‐limited media without added cellulose particles, nine of 13 species exhibited a pellet‐forming morphology. After a cultivation period of 6 days (Figure S3A–I), all pellet‐forming *Streptomyces* spp. exhibited a significantly smaller mean pellet Feret diameter in the condition with cellulose fibres compared to the unsupplemented condition (Figure 2). This was especially pronounced for *S. fradiae* , in which the presence of cellulose led to complete disintegration of the pellets, rendering pellet quantification impossible (Figure 2). This was also evident in the scattered‐light signal during cultivation, where a sharp drop was observed after approximately 1 day, which could indicate a dispersal event (Figure S3F). Analogously, stirred‐tank cultivations with *S. fradiae* have observed similar fragmentation of pellets under high shear conditions, in contrast to pellet formation in low‐shear conditions (Tamura et al. 1997). Additionally, some species also exhibited changes in pellet appearances. *S. cinereoruber* exhibited a morphological change from loose, open‐structured pellets to very dense ones. For *S. rimosus* , the distribution of pellet sizes changed markedly, with few very large pellets and a majority of small pellets with Feret diameters below 200 μm (Figure 2).

Taken together, cellulose particles influenced pellet size of *Streptomyces* spp. substantially. While most micro‐particle enhanced cultivations utilize particles below 50 μm, the cellulose fibres used in our study are around 150 μm in size (mesh 100), which is smaller than most pellets measured in this study but close to the undisturbed mean pellet size of several species (*S. cinereoruber, S. bobili, S. alboniger*).

Micro‐aggregates, such as pellets, grow radially, meaning they branch out and entanglement leads to dense cores and looser outer regions. Under low shear stress, larger aggregates collide and can merge into composite pellets (Zacchetti et al. 2018). Another important factor influencing pellet size is nutrient availability, as fragmentation is associated with the onset of the stationary phase in *Streptomyces*. This leads to pellets becoming less dense; outer filaments protrude and can be more easily detached as viable fragments (Zacchetti et al. 2018). A likely mechanism for pellet size reduction with the addition of cellulose fibres is an increase in local shear and abrasion, physically disrupting hyphal aggregation, which leads to smaller and more numerous pellets. We did not observe macromorphological changes for the four dispersed‐growing species, *S. collinus* , *S. nodosus* , *S. nitrosporeus* , and *S. venezuelae* .

### 3.2 Growth Parameters Do Not Correlate With Morphology

For evaluation of growth, both autofluorescence and scattered‐light signals were measured continuously during cultivation. However, for growth evaluation of *Streptomyces* spp. in the presence of cellulose particles, the measurement of scattered‐light will not be functional, since solid particles distort the measurement. To test whether the autofluorescence signal of *Streptomyces* can be used to monitor growth, the scattered‐light and autofluorescence signal of the cultivations without cellulose was analysed to detect the different growth phases and the resulting initial growth rates, which were quantitatively compared for the two measurements (Methods, Table S4). For most *Streptomyces* spp. (10 out of 13), initial growth behaviour in the absence of cellulose followed the same pattern across both measurements. The scattered‐light signal increases until a secondary substrate limitation occurs (dashed purple line); thereafter, a slower increase in signal intensity is observed until glucose limitation (dotted black line), after which the signal decreases or reaches a plateau (Figures S2A–D and S3A–I). For the three species *S. coeruleorubidus* , *S. nodosus* , and *S. alboniger* , the initial growth rate calculated from both measurements was different (Table S4). The autofluorescence signal exhibited a fluctuation during the initial growth phase, most likely due to a phosphate, nitrogen, or other secondary substrate limitation (Figures S2B and S3A–I). This has also been observed previously for *S. coelicolor* , where a phosphate limitation led to a fluctuation in the fluorescence signal at 450/520 nm (Finger et al. 2023; Palacio‐Barrera et al. 2022). The three species that exhibited significantly different rates between the two signals may produce pigments or autofluorescent molecules early in growth.

Since the initial growth rate *μ*_1_ determined from scattered‐light and autofluorescence did not differ significantly for most species, we next evaluated the influence of cellulose on initial growth. In the presence of cellulose, the initial growth rate from the autofluorescence, as a biological parameter for species fitness, did decrease significantly in five of 13 species (Table 1). Surprisingly, this was not strictly linked to morphological phenotype, as three species ( *S. filamentosus* , *S. avermitilis* , and *S. cinereoruber* ) were pelleted, whereas the other two ( *S. nodosus* and *S. collinus* ) exhibited a dispersed morphology. Pelleted species that exhibited a greater decrease in initial growth also tended to form more loosely structured pellets ( *S. avermitilis* , *S. filamentosus* ). This might be because cellulose fibres exert stronger effects on dispersed mycelia or on looser pellets than on dense pellets, where they cannot fragment the pellets as easily. In our study, the addition of cellulose primarily reduced growth rates. Other solid particles, like glass beads, often have opposite effects. For example, in microtiter plate cultivations of *S. coelicolor* with 3 mm glass beads, *μ*_max_ was increased, as well as product titers, while pellet size decreased (Sohoni et al. 2012).

**TABLE 1:** Comparison of growth parameters from the autofluorescence signal between the cultivation conditions (−/+ cellulose) for all 13 *Streptomyces* spp.

Morphology	Species	Condition: Cellulose present	Mean *μ*_1_	*p*	Mean AUC	*p*
Pellet forming	*S. coeruleorubidus*	−	0.097 ± 0.004	0.110	1238 ± 121	0.431
		+	0.090 ± 0.003		1326 ± 62	
	*S. cinereoruber*	−	0.209 ± 0.052	0.044	1425 ± 11	0.193
		+	0.052 ± 0.012		1567 ± 105	
	*S. filamentosus*	−	0.280 ± 0.014	0.003	2714 ± 82	0.044
		+	0.158 ± 0.005		2498 ± 57	
	*S. avermitilis*	−	0.113 ± 0.003	0.001	1537 ± 101	0.006
		+	0.071 ± 0.005		2033 ± 92	
	*S. bobili*	−	0.142 ± 0.009	0.562	1534 ± 12	0.0001
		+	0.138 ± 0.003		2447 ± 31	
	*S. fradiae*	−	0.277 ± 0.008	0.171	2501 ± 152	0.003
		+	0.243 ± 0.024		8836 ± 648	
	*S. albofaciens*	−	0.329 ± 0.002	0.913	1752 ± 18	0.001
		+	0.331 ± 0.02		3431 ± 90	
	*S. rimosus*	−	0.214 ± 0.003	0.149	1783 ± 40	0.001
		+	0.203 ± 0.007		2432 ± 9	
	*S. alboniger*	−	0.166 ± 0.002	0.914	3711 ± 383	0.014
		+	0.166 ± 0.003		5174 ± 278	
Dispersed	*S. venezuelae*	−	0.235 ± 0.013	0.045	2982 ± 1172	0.060
		+	0.195 ± 0.002		5944 ± 346	
	*S. collinus*	−	0.152 ± 0.004	0.0001	769 ± 12	0.845
		+	0.093 ± 0.003		760 ± 59	
	*S. nodosus*	−	0.060 ± 0.001	0.008	1234 ± 118	0.193
		+	0.024 ± 0.005		1076 ± 27	
	*S. nitrosporeus*	−	0.070 ± 0.001	0.106	1635 ± 34	9.83 × 10^−6^
		+	0.081 ± 0.006		2599 ± 35	

*Note:* The initial growth rate *μ*_1_ differed significantly between the conditions for five species, while the area under the curve (AUC) differed significantly for eight species (*t*‐test, *α* = 0.05 indicated by orange and green shaded area, *n* = 3).

Additionally, the AUC of the autofluorescence measurement was used as an estimate to compare growth behaviour over the entire cultivation period (Table 1). Although initial biomass formation was not affected by the addition of cellulose in eight species, AUC increased significantly in seven species, while only *S. filamentosus* showed a significant decrease with the cellulose fibres. The change in AUC was more pronounced for pellet‐forming species. The only species with a dispersed morphology that was affected by cellulose fibres was *S. nitrosporeus* (Figure S2D). While the AUC is not significantly different, *S. venezuelae* also exhibits increased autofluorescence after secondary nutrient limitation, which is noticeably higher with the addition of cellulose (Figure S2A). The integrals of two curves, however, can be quantitatively similar yet differ in their trajectories. This is evident for *S. cinereoruber* , where curve trajectories differ markedly across conditions, yet the AUCs are not significantly different (Figure S3B). Compared with scattered‐light measurements, autofluorescence measurements provide additional information because nutrient limitations can be visible as fluctuations in the fluorescence signal. Interestingly, once the carbon source is limited, as visible through a decrease in scattered‐light, the autofluorescence curve further increases for many of the *Streptomyces* spp. (*S. coeruleorubidus, S. avermitilis, S. filamentosus, S. nitrosporeus, S. nodosus, S. fradiae*) (Figures S2A–D and S3). This effect has been previously observed for *S. coelicolor* A3(2) at an autofluorescence wavelength pairing of 483/520 nm (Finger et al. 2023) and for *S. coeruleorubidus* at 400/470 nm (Brauneck et al. 2025), where an increase in the autofluorescence signals coincided with glucose depletion, which was confirmed through a characteristic drop in the oxygen transfer rate. It has been suggested that this increase in autofluorescence may be related to the oxidation of flavins under carbon‐source limitation, as flavins are more fluorescent in their oxidized state (Surre et al. 2018). Since carbon limitation affects autofluorescent molecules, AUC comparisons of the autofluorescence between conditions for the same species can provide insights into metabolic changes. This is supported by the observation that eight of 13 species exhibited significant changes in AUC in the presence of cellulose. Taken together, these observations indicate that autofluorescence is a useful proxy for monitoring growth dynamics in the presence of cellulose particles. The signal is also influenced by physiological changes, such as nutrient limitation, pigment formation, and cellular redox state; however, it may be affected by these factors independently of biomass formation.

### 3.3 Many Secondary Metabolites Decrease With the Addition of Cellulose Particles

**FIGURE 3:**
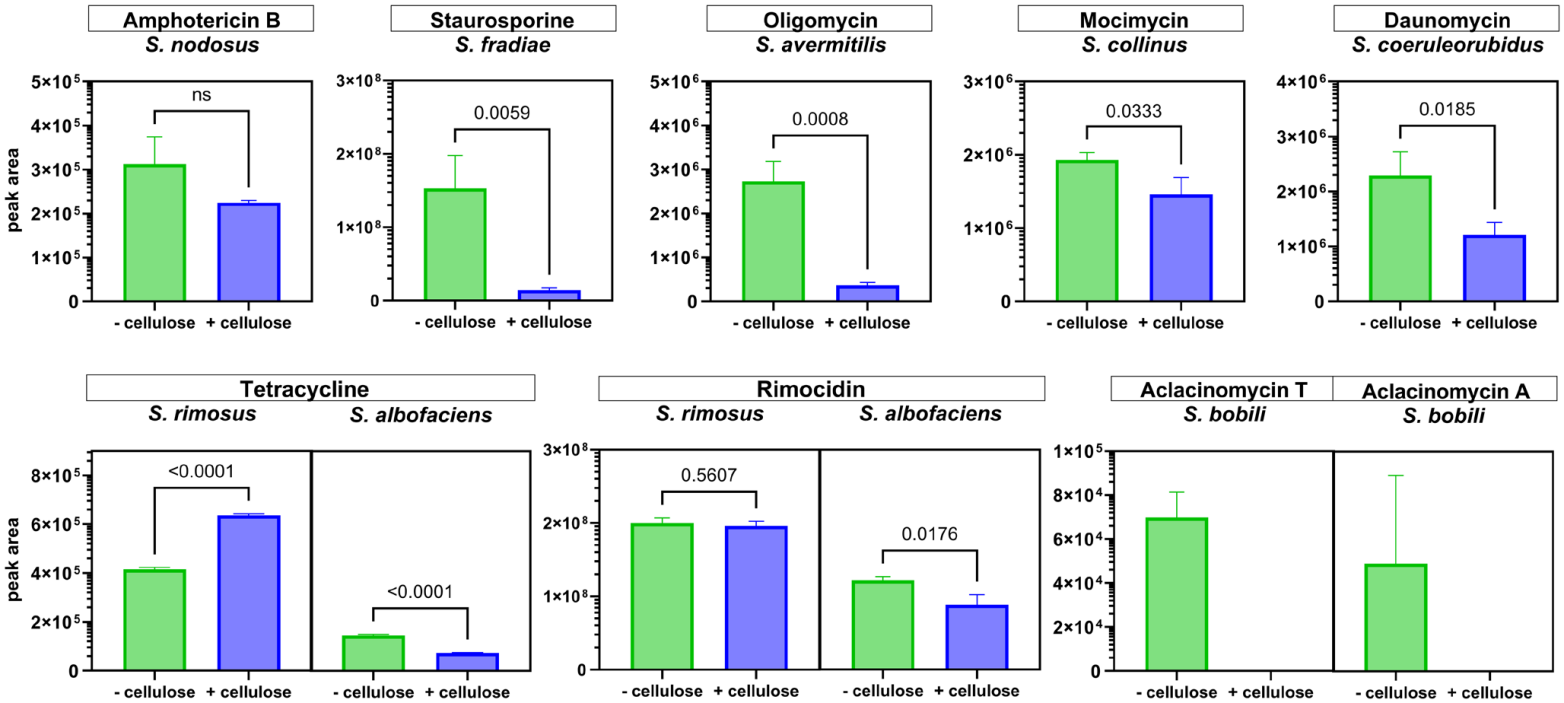
Comparison of mean peak area between conditions (−/+ cellulose) for annotated natural products. Annotation scores are given in Table S5. Unpaired two‐sided *t*‐tests were used for compounds detected in both conditions (*α* = 0.05). Aclacinomycin T and A were detected in the (−) cellulose condition but were below the detection limit in the (+) cellulose condition.

Many of the tested *Streptomyces* spp. are well known for producing natural products, such as oligomycin from *S. avermitilis* or staurosporine from *S. fradiae* (Guan et al. 2019; Lin et al. 2009). Therefore, we first evaluated the impact of cellulose addition on known secondary metabolites by performing a targeted metabolite analysis of potential candidates. After selecting metabolites that could be annotated with high confidence scores (Table S5), we tested whether the mean peak areas differed between the cultivation with and without cellulose fibres (Figure 3).

The dispersed growing species exhibited little change in natural product production in the presence of cellulose, similar to the lack of morphological changes. *S. nodosus* , whose AUC did not change significantly, also did not exhibit a significant decrease in the amphotericin B peak area. While the AUC did not differ between conditions, *S. collinus* produced 1.3‐fold less mocimycin (kirromycin) (Figure 3). In the case of pellet‐forming *Streptomyces*, the decrease in pellet size resulted in lower abundances of natural products. The following secondary metabolites exhibited significantly smaller mean peak areas in the cellulose conditions: oligomycin (*S. avermitilis*, seven‐fold decrease), staurosporine (*S. fradiae*, eleven‐fold decrease), daunomycin (*S. coeruleorubidus*, two‐fold decrease), and rimocidin (*S. albofaciens*, 1.3‐fold decrease). *S. rimosus* also produced the polyene macrolide rimocidin, but the mean peak area did not change significantly with the addition of cellulose. Notably, this species is the only pelleted one where the investigated natural products did not decrease in the presence of cellulose, despite a decrease in pellet size. We further found that the tetracycline peak area was significantly increased by a factor of 1.5 with the addition of cellulose in this species (Figure 3). A similar behaviour was observed in a study by Scigaczewska et al., in which the addition of talc microparticles did not alter rimocidin concentrations in *S. rimosus* , but led to an up to 9‐fold increase in oxytetracycline (Scigaczewska et al. 2024). The observation that secondary metabolite production is regulated very differently across species is supported by the finding that the tetracycline concentration in *S. albofaciens* was significantly reduced by the addition of cellulose (1.9‐fold decrease). We also identified two anthracyclines in *S. bobili* grown in the absence of cellulose. But both aclacinomycin T (aklavin) and aclacinomycin A (aclacur) were below the detection limit in the cultivation with cellulose.

Interestingly, this contrasts prior microparticle‐enhanced cultivations, where the addition of talc had a positive effect on the formation of natural products in six out of nine tested *Streptomyces* spp. (Kuhl et al. 2021). The mechanisms underlying particle‐enhanced cultivation are strongly dependent on particle size. Microparticles (< 50 μm) are thought to interfere with spore agglomeration and become incorporated into developing pellets, resulting in a loosened pellet core and improved nutrient and oxygen supply to the hyphae. In *Aspergillus niger* cultivations, microparticles, such as talc or glass particles, modify morphological parameters, including pellet size, compactness, and fragmentation, by changing local shear and nucleation, which in turn alter oxygen and nutrient gradients inside pellets (Dinius et al. 2024). It was found that smaller glass particles, around the size of fungi spores, have a positive effect on product titers by decreasing the total hyphal fraction of the pellets, thereby decreasing pellet density and enabling better nutrient supply (Dinius et al. 2024). In contrast, larger particles, such as glass or ceramic beads, primarily act through repeated particle–hypha and particle–particle collisions, causing abrasion and mechanical stress, while bead‐induced compression has also been proposed to improve mass transfer by expelling liquid from the pellet interior (Schrader et al. 2023). The cellulose fibres used in our study are around 150 μm in size (mesh 100); *Streptomyces* hyphae filaments are much smaller than fungal hyphae, and we inoculated our cultures with dispersed mycelia, not spores. These methodological differences might explain the reducing effect of cellulose on product titers in this study. Although the cellulose fibres are too large to become incorporated into the pellet core and are therefore unlikely to loosen it internally, they may still impose shear and abrasion forces on the pellet surface, albeit to a lesser extent than glass or ceramic particles due to their lower density and softer nature. Consequently, the observed reduction in pellet size may result from repeated low‐energy particle–hypha contacts that remove loosely attached peripheral hyphae or interfere with the aggregation and fragmentation dynamics that determine *Streptomyces* pellet size (Zacchetti et al. 2018). Besides particle size, physicochemical properties such as surface charge, hydrophobicity, particle density, and material composition have been shown to influence particle–cell interactions in particle‐enhanced cultivations (Laible et al. 2022). Compared to inorganic particles such as talc or Al_2_O_3_, cellulose fibres possess different surface chemistry and a lower density, which may reduce their interaction with the biomass and contribute to their distinct effects on morphology.

Additionally, many microparticle studies in filamentous fungi employ spore inocula, where particles interfere with early spore aggregation and subsequent pellet formation (Dinius et al. 2024). In contrast, our cultivations were inoculated with dispersed mycelia instead of spores, which might hinder the process of incorporating the cellulose fibres because pre‐existing cell clumps and newly branching hyphae are not entangling with each other as much as freshly germinating spores would. Consequently, the mechanisms proposed for fungal spore‐based microparticle‐enhanced cultivations cannot be directly transferred to the present study.

Taken together, there is a trend towards a decrease in natural product production in the presence of cellulose for the tested *Streptomyces* spp., despite a decrease in pellet Feret diameter that could suggest a higher productivity. To understand the underlying mechanism of this trend and to identify global effects of the cellulose addition on metabolic profiles, we applied untargeted metabolomics next.

### 3.4 Species Identity Is the Main Driver of Overall Metabolic Differences, Which, in the Absence of Cellulose, Correlate With Biosynthetic Potential

**FIGURE 4:**
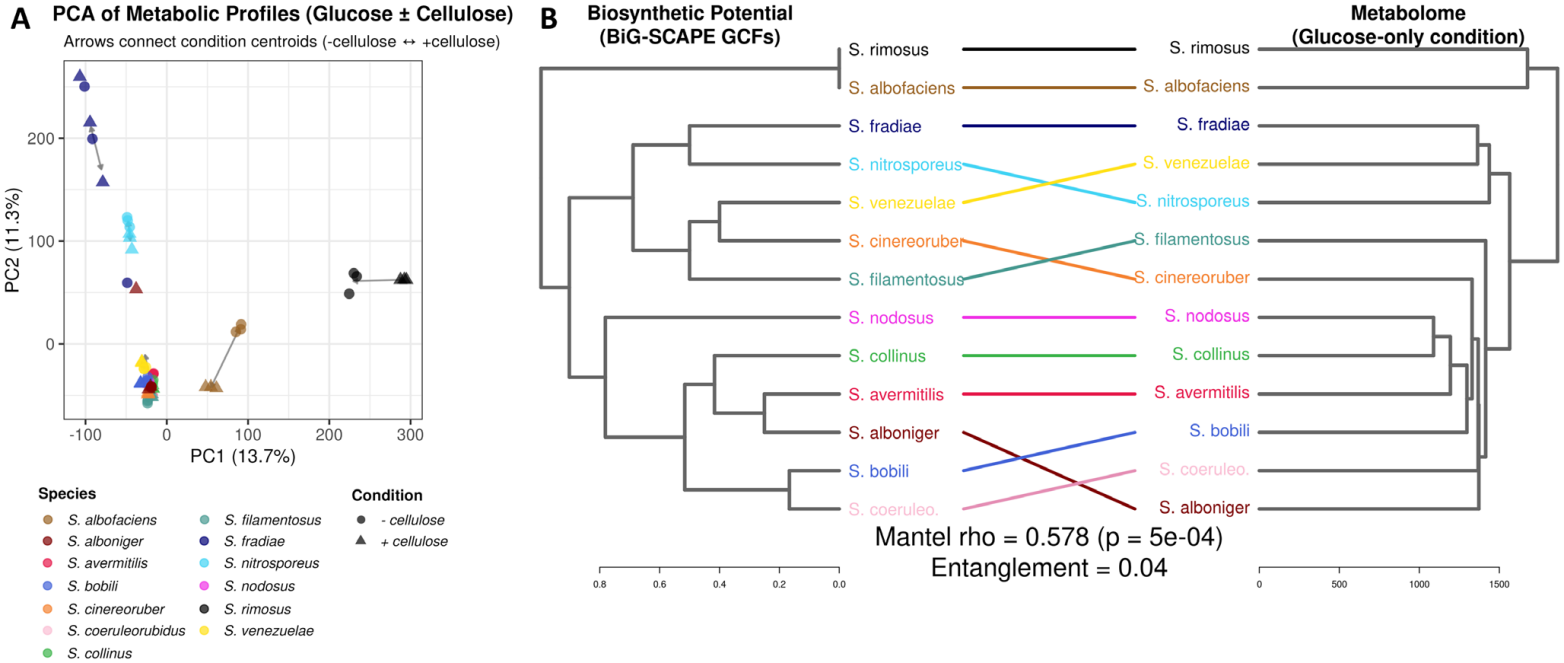
Results of untargeted metabolomics. (A) Principal component analysis (PCA) of the metabolic features of all 13 *Streptomyces* spp. reveals that the addition of cellulose only causes minor differences between samples of some species. Arrows connect condition centroids. (B) Global metabolic differences between species reveal that the metabolome closely represents the biosynthetic potential of *Streptomyces* spp., Tanglegram comparing the biosynthetic tree (left, average‐linkage clustering on Jaccard distances of shared Gene Cluster Families) with the metabolomic tree from the glucose‐only condition (right, average‐linkage clustering on Euclidean distances of log2‐transformed, PQN‐normalized peak areas); tree similarity was quantified using a Mantel test with Spearman rank correlation. Colours indicate species (*n* = 3 per species and condition).

To gain a broader view of metabolic differences between species and to test whether any species shared a global metabolic response to the addition of cellulose particles, we used untargeted metabolomics. We performed a principal component analysis (PCA) on the PQN‐normalized abundances of metabolic features to obtain an overview of metabolic differences between the species and conditions (Figure 4A). The species identity appeared to be the primary driver of variance, while the addition of cellulose only played a minor role for the separation in the PCA. However, for some species, particularly for *S. rimosus* and *S. albofaciens* , distinct clusters indicating large metabolic changes upon the addition of cellulose are observable.

A comparison of the phylogenetic relationship between the species (Figure S4) indicated that metabolic differences might reflect phylogeny, which is probably linked to species biosynthetic potential. Since most *Streptomyces* are known to produce highly specialized secondary metabolites, we performed a genome‐mining analysis of biosynthetic gene clusters and tested the relationship with the metabolome under undisturbed conditions on glucose without cellulose (Figure 4B). The significant Mantel correlation (Spearman *ρ* ≈ 0.578, *p* < 0.0005) between the biosynthetic potential (represented by non‐singleton gene cluster families) and the metabolome supports the hypothesis that the distinct metabolic clustering of species in the PCA is linked to their unique BGC repertoires. The low entanglement (0.04) indicates that species with similar genomic repertoires cluster together metabolically. Although many species only share few BGCs, the pairwise distance relationships are preserved: species that are phylogenetically and biosynthetically closer (e.g., *S. rimosus* and *S. albofaciens* ) produce metabolomes that are more chemically similar. This moderate yet significant correlation suggests that genomic potential accounts for a substantial fraction of the metabolic variance under glucose‐only conditions (no cellulose added). To confirm robustness, the analysis was repeated on multiple metabolic subsets and also with phylogenetic distance statistically controlled by a partial Mantel test; the correlation remained significant in all cases (Table S6).

In the presence of cellulose, the metabolome no longer correlates with the biosynthetic potential of the species (Mantel ρ = 0.176, *p* = 0.146) (Figure S5). We tested and found no significant correlation across three definitions of the response to cellulose (all features, significantly changed features, and top‐variable features, see Methods). Given the loss of correlation between metabolome and BGC repertoire upon the addition of cellulose, a metabolic switch might have occurred in response to the cellulose particles. The species that exhibit stronger metabolic responses do not seem to share a similar BGC repertoire.

### 3.5 Cellulose Addition Leads to a Decrease in Peptides Highly Abundant Across Species and Species‐Specific Metabolic Changes

Since particle stress can alter gene expression and the metabolic state of *Streptomyces* spp. (Kuhl et al. 2020), it is crucial to understand metabolome‐wide changes under specific stress conditions and whether these responses are species‐specific or can be placed into a broader phylogenetic context. We therefore used a linear model adjusted for species identity to determine metabolites that were significantly differentially abundant between the two conditions. Additionally, the metabolites were annotated to the chemical class level using CANOPUS within SIRIUS (Dührkop et al. 2021). This provided a comprehensive insight into the metabolic changes that occurred with the addition of cellulose particles during *Streptomyces* spp. cultivation.

**FIGURE 5:**
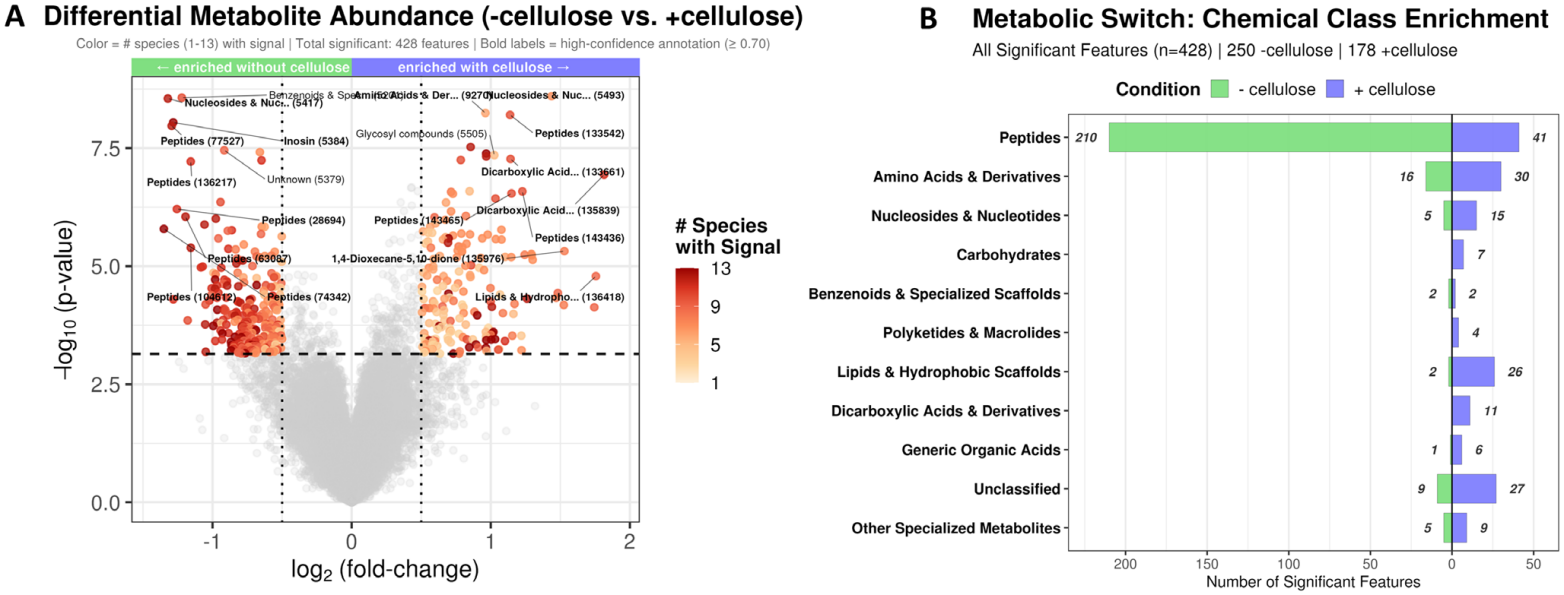
Identification and classification of differentially abundant metabolites. (A) Volcano plot of features with significantly altered abundance (|log2(fold change)| ≥ 0.5, FDR‐adjusted *p* < 0.05) for conditions without (left) and with cellulose (right). Labels show the ten highest‐impact features per condition, ranked by |log2 FC| × −log10(*p*). The colour scale indicates the number of species in which the metabolite exhibited a robust signal (which is double the noise value). (B) Chemical class annotation of all differentially abundant metabolites for each condition according to CANOPUS.

The model identified a core set of 428 metabolites that differed significantly in abundance between the two conditions (250 enriched without cellulose, 178 enriched with cellulose; Figure 5A). Metabolites significantly enriched in the absence of cellulose (log2 fold‐change < −0.5) are common to a greater number of species, whereas metabolites induced in the presence of cellulose (log2 fold‐change > 0.5) show a more heterogeneous response. Cellulose‐associated metabolites also exhibit a broader chemical range, with most metabolites shared by six to eight species (Figure 5A). Interestingly, in terms of chemical classes, a lot of peptides, which are widespread and can be detected in up to 13 species, decrease in abundance upon the addition of cellulose.

After 6 days of cultivation, all 13 *Streptomyces* spp. had reached the stationary phase before endpoint samples were collected. This leads us to hypothesize that all species were already experiencing sporulation and programmed cell death to provide nutrients for sporulating cells. This would be consistent with the higher abundance of peptides in the condition without cellulose (Figure 5B), where cells were already lysing and thus releasing proteolytic peptides. These increased peptide abundances in some strains might be linked to a change in cell cycle progression. In *Streptomyces*, nutrient starvation often leads to morphological differentiation and secondary metabolite production, which is supported by nutrients released by autolysis of substrate mycelium (Romero‐Rodriguez et al. 2018). This nutrient‐scavenging, or programmed cell death, is observed in sporulating bacteria, such as *Bacillus subtilis* and *Streptomyces griseus* , in which sporulating cells release a killing factor and a signalling protein that cause adjacent cells to lyse rather than sporulate (Birko et al. 2009; González‐Pastor et al. 2003). A direct link between programmed cell death and antibiotic production has been demonstrated, with cell wall–derived compounds (such as N‐acetylglucosamine) identified as a key signalling molecule initiating development and antibiotic production in *Streptomyces* (Rigali et al. 2006, 2008).

In the presence of cellulose, metabolites with increased abundance compared to cultivation without cellulose included amino acids and derivatives, lipids, hydrophobic scaffolds, and unclassified metabolites (Figure 5B). These changes are likely due to the increased mechanical stress introduced by the cellulose. The cellulose fibres further damage dying nutrient‐starved cells, spilling their contents into the medium or altering their metabolic profiles in response. This might explain the decrease in the number of induced peptides when compared to the non‐cellulosic conditions. Still, this compound class remains as the dominant fraction among all the significantly increased metabolites under cellulose conditions (41 out of 178), and the increase of amino acids and derivatives (30 out of 178) might be degradation products of those peptides. Additionally, cell membrane components might be broken down into lipids and hydrophobic scaffolds (26 out of 178), and cell contents, such as nucleosides and nucleotides (15 out of 178), are increasingly found in the analysed sample. In comparison to the condition without cellulose, a large fraction of metabolites was unclassified (27 out of 178) under cellulose conditions.

**FIGURE 6:**
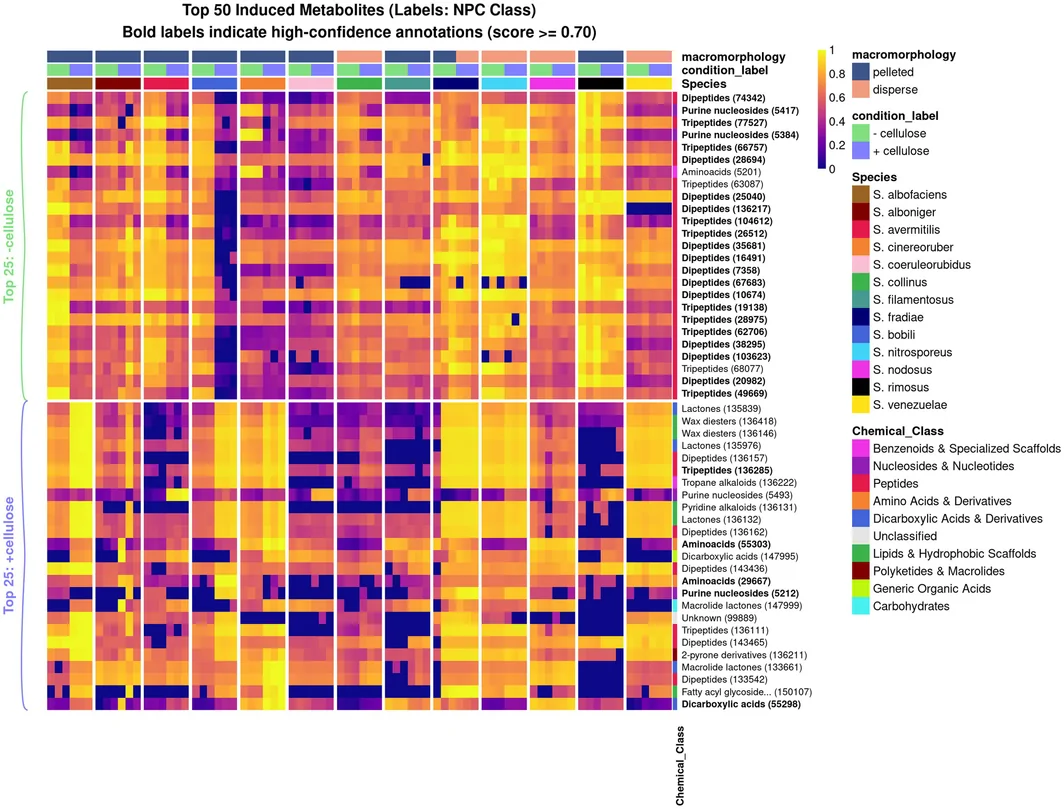
Heatmap showing the abundance of the top 25 features that were up‐regulated without (−) cellulose (top half) and with (+) cellulose (lower half). Colours indicate species identity, morphological type, and specific chemical subclass annotations to highlight structural diversity. Heatmap colours represent row‐wise min‐max scaled PQN peak areas (0 = lowest peak area detected in that row, 1 = highest). Metabolite annotations on the right, bold labels indicate high annotation confidence (CANOPUS NPClassifier class probability ≥ 0.70).

A closer examination of the top 25 induced metabolites in each condition revealed that the most prominent global metabolic changes were shared by three to four species (Figure 6). *S. albofaciens* , *S. bobili* , and *S. avermitilis* exhibited the most prominent decrease of peptides with the addition of cellulose, while the fold‐changes of these metabolites for other species were smaller. To exclude cellulase activity as a potential factor causing these metabolic differences, filter paper assays were performed; no cellulase activity was detected in *S. avermitilis, S. bobili, S. coeruleorubidus*, or *S. albofaciens* (Supporting Information, Material and Methods, Figure S6A,B). Metabolites that generally increased in the presence of cellulose were strongly increased in *S. albofaciens* , *S. bobili* , and *S. cinereoruber* (Figure 6). This indicates that out of nine pellet‐forming species, four exhibit prominent changes, but no dispersed growing species exhibits strong metabolic responses. All four species showed a significant decrease in pellet size, and, morphologically, these species also formed pellets on the looser end of the spectrum. Except for *S. cinereoruber* , where a drastic change from looser pellets without cellulose to denser pellets with cellulose occurred. Generally, *Streptomyces* spp. that exhibit denser pellets appear to be metabolically less affected by the addition of cellulose to the cultivation medium. The change of metabolic profiles for species with looser pellets might be related to an increase in additional cell rupture instead of programmed cell death. Ruptured cells release cellular building blocks into the medium that can then be utilized as nutrients by other starving cells, which delays cell cycle progression. The *Streptomyces* life cycle involves two sequential programmed cell death events. The first transforms the compartmentalized mycelium into a multinucleated vegetative form, and the second accompanies the development of aerial mycelium formation (Manteca et al. 2011). It has been shown that the external supply of phosphorylated sugars under phosphate limitation can delay the occurrence of the second round of programmed cell death, leading to a vegetative state and inhibiting the production of antibiotics (Tenconi et al. 2012).

Whether the increase in amino acids, lipid scaffolds, and nucleosides upon addition of cellulose is caused by a purely mechanical process or a metabolic adaptation is not clear. Transcriptomic analysis of *Streptomyces albus* showed that upon talc addition, metabolic pathways for branched amino acids, polyketide metabolism, and biosynthesis of secondary metabolites were increased during the growth phase (Kuhl et al. 2020). Four features that were increased in the presence of cellulose have been putatively annotated as polyketides, indicating a differential pathway regulation. Taken together with the high number of increased metabolites of unknown identity, the cultivation with cellulose particles might have led to metabolically changed responses or triggered de novo synthesis of novel metabolites.

## 4 Conclusion

Multiple secondary metabolites, such as antibiotics, have been developed from *Streptomyces*, and various studies have shown that increased hydrodynamic shear stress in industrial bioreactors can affect antimicrobial production. To investigate the effect of shear stress mediated by the addition of cellulose particles on less‐studied *Streptomyces* spp., we evaluated the impact of cellulose on growth behaviour, morphology, and secondary metabolite profiles of 13 species. Cellulose reduced pellet size and predominantly lowered the amounts of several natural products detected via LC–MS, likely due to increased cell degradation during the stationary phase under elevated mechanical stress. Our results reveal a complex, species‐dependent response: individual metabolites were variably up‐ or downregulated, with stronger effects observed in pelleted morphotypes. In the absence of cellulose, the metabolome was strongly linked to the genetically encoded biosynthetic potential, whereas the cellulose‐associated response was no longer structured by phylogeny. This pattern is consistent with a broadly shared response to cellulose‐associated stress that overlays species‐specific biosynthetic fingerprints, although endpoint sampling cannot fully distinguish active metabolic response from increased release of metabolites from damaged cells.

The here presented analysis showed that *Streptomyces* spp. alone undergo a profound metabolic reprogramming with the addition of cellulose particles. Although the addition of cellulose led to the downregulation of many known metabolites in our experiments, we also observed clear instances of upregulated metabolites, including previously unknown compounds. These findings suggest that the potential of cellulose supplementation to induce novel metabolites is highly species and context‐dependent. How the cellulose supplementation compares to other established cultivation‐enhancing particles and whether the effects observed here would be scalable to stirred‐tank bioreactors remains to be evaluated experimentally in the future. Cellulolytic fungi, on the other hand, can degrade cellulose and can slowly release glucose over time. Moreover, fungal cell wall components, such as N‐acetylglucosamine, can potentially induce the production of secondary metabolites (Martin et al. 2011). Furthermore, since secondary metabolite formation is strongly linked to carbon limitation in *Streptomyces*, it appears feasible that a cellulolytic fungus, such as *Trichoderma reesei*, facilitating a slow release of glucose could be exploited to create a dependency of *Streptomyces* spp. on the fungus and thus induce the formation of natural products. The here characterized *Streptomyces* spp., and especially their specific response to particle‐induced mechanical stress by cellulose, thus represent an ideal basis for more sophisticated co‐culture experiments in the future to isolate and identify new natural products.

## Author Contributions

**Jethro L. Hemmann:** conceptualization, data curation, methodology, supervision, writing – review and editing. **Gesa Brauneck:** conceptualization, writing – review and editing. **Ivan Schlembach:** conceptualization, funding acquisition, methodology, supervision, writing – review and editing. **Bashar Al‐Smadi:** conceptualization, data curation, formal analysis, methodology, writing – original draft, writing – review and editing. **Miriam A. Rosenbaum:** conceptualization, funding acquisition, project administration, resources, supervision, writing – review and editing. **Ana M. Palacio‐Barrera:** conceptualization, supervision, writing – review and editing. **Dorothea M. Schütterle:** conceptualization, data curation, investigation, methodology, writing – original draft, writing – review and editing. **Jorgen Barsett Magnus:** conceptualization, writing – review and editing. **Sascha Schäuble:** conceptualization, methodology, supervision, writing – review and editing. **Gianni Panagiotou:** conceptualization, supervision, writing – review and editing.

## Funding

This work was supported by the Deutsche Forschungsgemeinschaft (priority program: SPP2170, project number: 427899901), the Cluster of Excellence ‘Balance of the Microverse’ under Germany's Excellence Strategy (EXC 2051—project ID 390713860). J.L.H. was supported by the Free State of Thuringia and the European Social Fund Plus [2024FGR0017].

## Conflicts of Interest

The authors declare no conflicts of interest.

## Supporting information


**Table S1:** Channel settings of the Biolector I during cultivations.
**Table S2:** Filter settings of the Keyence BZ‐X810 microscope during image acquisition.
**Table S3:** DSM numbers and NCBI accession numbers of cultivated *Streptomyces* species. Solid line indicates different morphological types.
**Figure S1:** Results of LC–MS/MS quality control metrics. (Median MS1 error in PPM, median retention time shift, total number of MS2 spectra per sample, median log2 intensity per sample, and number of detected features).
**Table S4:** Comparison of growth rates derived from autofluorescence and scattered‐light signal, for cultivations without cellulose. For 10 of the 13 species, the initial growth rate, based on the autofluorescence and scattered‐light signals, is not significantly different (paired *t*‐test, *α* = 0.05) during cultivation without cellulose addition.
**Table S5:** Detailed description of natural products and their annotation by SIRIUS.
**Table S6:** Additional Mantel tests confirming the robustness of BGC and metabolome correlation.
**Figure S2:** Growth curves of scattered‐light (SL) without cellulose (grey), autofluorescence (AF) without cellulose (green), and with cellulose (blue) for dispersed *Streptomyces* spp. (*n* = 3 per species and condition). Dashed purple line indicates presumed secondary substrate limitation, dotted black line indicates presumed glucose limitation. (A) *S. venezuelae* , (B) *S. collinus* , (C) *S. nodosus* , (D) *S. nitrosporeus*.
**Figure S3:** Growth curves of autofluorescence (AF) without cellulose (green) and with cellulose (blue), scattered‐light (SL) without cellulose (dark grey) for pelleted *Streptomyces* spp. (*n* = 3 per species and condition). Dashed purple line indicates presumed secondary substrate limitation, dotted black line indicates presumed glucose limitation. (A) *S. coeruleorubidus* , (B) *S. cinereoruber* , (C) *S. filamentosus* , (D) *S. avermitilis* , (E) *S. bobili* , (F) *S. fradiae*, where the scattered‐light measurement (light brown) with cellulose shows a drop, which could potentially be a dispersal event of the pellets. (G) *S. albofaciens* , (H) *S. rimosus*, (I) *S. alboniger* .
**Figure S4:** Phylogenetic tree of *Streptomyces* spp. created with autoMLST. The outgroup was *Micromonospora inositola* .
**Figure S5:** Response tanglegram comparing the biosynthetic potential vs. the metabolic response (+ cellulose minus −cellulose). The increase in entanglement (0.10 vs. 0.04 at glucose baseline) visually demonstrates a weaker correspondence between biosynthetic potential and the cellulose‐associated response than in the glucose‐only metabolome.
**Figure S6:** Evaluation of cellulase activity showed no relevant activity in *S. albofaciens, S. avermitilis, S. bobili*, and *S. coeruleorubidus*. A: After 6 days of shake flask cultivation (10 mL, 200 rpm, 50 mm shaking diameter) in LNP medium containing 30 g L^−1^ glucose and 30 g L^−1^ cellulose, none of the *Streptomyces* cultivations exceeded the theoretical OTR integral limit (1000 mmol L^−1^), corresponding to complete respiration of supplied glucose. Comparatively, *Trichoderma reesei* RUT‐C30, which was grown on 5 g L^−1^ glucose and 30 g L^−1^ cellulose, substantially exceeded the theoretical OTR integral limit (166.5 mmol L^−1^). B: Filter paper assays showed that, in comparison to supernatant from positive control (*Trichoderma reesei* RUT‐C30), supernatants from *Streptomyces* species did not cause glucose release from the cellulose filter paper after 1 h of incubation at 50°C.

## Data Availability

The untargeted LC–MS/MS data (.mzML format) and processed feature quantification tables have been deposited to the MassIVE repository with ReDU‐compatible metadata to support FAIR data principles (MSV000101904). Genomic reference sequences were retrieved from NCBI using the accessions listed in Table S3.
