# Supplementary material for: Influence of Cellulose Particles on Streptomyces spp. Growth, Morphology, and Metabolite Spectrum
Source: Microb Biotechnol. 2026 Sep 16;19(9):e70445. doi: 10.1111/1751-7915.70445 (PMC13583446; doi:10.1111/1751-7915.70445)
Supplement: Supplementary file 1 [file MBT2-19-e70445-s001.docx]

Supporting Information: Influence of cellulose particles on *Streptomyces* spp. growth, morphology, and metabolite spectrum

**Dorothea M. Schütterle^1,4^, Bashar Al-Smadi^2^, Jethro L. Hemmann^3^, Gesa Brauneck^5^, Ana M. Palacio-Barrera^1,6^, Ivan Schlembach^1^, Sascha Schäuble^2^, Jorgen Barsett Magnus^5^, Gianni** **Panagiotou^2,4,6^, Miriam A. Rosenbaum^1,4,6*^**

^1^Bio Pilot Plant, Leibniz Institute for Natural Product Research and Infection Biology – Hans-Knöll-Institute, Jena, Germany

^2^Department of Microbiome Dynamics, Leibniz Institute for Natural Product Research and Infection Biology – Hans-Knöll-Institute, Jena, Germany

^3^Metabolomics-guided Natural Product Discovery, Leibniz Institute for Natural Product Research and Infection Biology – Hans-Knöll-Institute, Jena, Germany

^4^Faculty of Biological Sciences, Friedrich-Schiller-University Jena, Jena, Germany

^5^AVT – Biochemical Engineering, RWTH Aachen University, Aachen, Germany

^6^Cluster of Excellence Balance of the Microverse, Friedrich-Schiller-University Jena, Jena, Germany

*** Correspondence:**
Miriam A. Rosenbaum
Miriam.rosenbaum@leibniz-hki.de

# 1 Supplemental Material and Methods

1.1 **LC-MS/MS Quality control measures**

To assess the technical stability of the LC-MS/MS instrument and feature alignment, quality control metrics were derived from the mapping of MS/MS spectra to the final feature list. These include the median precursor mass (MS1) error in PPM, the median retention time shift, the total number of MS2 spectra per sample, the median log2 intensity per sample, and the number of detected features. The distributions of these metrics were compared between the two groups: samples cultivated on glucose alone and on glucose with cellulose. Results show no significant differences between groups (Figure 1).

1.2 **Cellulase activity tests**

To verify the absence of cellulase formation and cellulose degradation, filter paper assays were conducted using culture supernatants. Four Streptomyces species (*S. albofaciens, S. avermitilis, S. bobili*, and *S. coeruleorubidus*) were initially cultivated in straight-rim flasks containing 10 ml of M79 complex medium (Prauser and Falta 1968). The cellulolytic fungus *Trichoderma reesei* RUT-C30 mCherry served as a positive control and was cultivated under identical conditions. Pre-cultures (10% v/v) were inoculated into straight-rim flasks containing 5 ml of LNP medium (30 g L⁻¹ glucose) and 15 sterile stainless-steel beads (2–2.5 mm) to facilitate dispersion of biomass. Cultures were incubated at 28 °C, 140 rpm for 48 h. The collected biomass was dispersed, and a stock suspension of OD₂ was prepared as described in Section 2.3. From this stock, 250 ml unbaffled shake-flask cultivations containing 10 ml of LNP medium (30 g L⁻¹ glucose and 30 g L⁻¹ cellulose) were inoculated to an initial OD of 0.1. Triplicate cultures were incubated at 30 °C and 200 rpm in a Kuhner shaker (ISF1‑X, Adolf Kuhner AG, Birsfelden, Switzerland) with a 5 cm shaking diameter. Exhaust gas analysis and determination of oxygen and carbon dioxide transfer rates (OTR and CTR) were performed with a Kuhner TOM system (Adolf Kuhner AG, Birsfelden, Switzerland). Cultures were aerated with compressed air (11 ml min⁻¹) and each measuring cycle lasted 18 min, of which 5 min were used for signal acquisition.

After six days of incubation, 1 ml of culture broth was harvested, centrifuged at 13,000 rpm for 2 min, and the resulting supernatant was stored at –20 °C until analysis. Filter paper assays were performed in 96‑well PCR microplates. Filter paper disks (Whatman No. 1) were placed in each well, followed by the addition of 40 µl sodium citrate buffer (75 mM, pH 4.8) and 20 µl of the sample. Plates were sealed, vortexed, centrifuged, and incubated at 50 °C for 1 h in a Mastercycler. A glucose standard series (10, 8, 6, 4, 2, 1 g L⁻¹) was included. Each supernatant was tested in triplicate with one blank (no filter paper). Following incubation, plates were placed on ice to terminate cellulase activity. Then, 120 µl of DNS reagent (prepared from 1416 ml water, 10.6 g dinitrosalicylic acid, 19.8 g NaOH, 306 g potassium‑sodium tartrate, and 8.3 g sodium metabisulfite) was added, mixed, centrifuged, and incubated at 95 °C for 10 min. Absorbance was measured at 540 nm using a plate reader (CLARIOstar, BMG LABTECH GmbH, Germany), and released glucose concentrations were calculated accordingly.

1.3 **Tables**

**Table S1: Channel settings of the Biolector I during cultivations**

| **Biolector channel** | **scattered-light** | **autofluorescence** |
| --- | --- | --- |
| Filter module | Biomass | HPTSII |
| Excitation wavelength [nm] | 620 | 450 |
| Emission wavelength [nm] | 620 | 520 |
| Gain | 15 | 100 |

**Table S2: Filter settings of the Keyence BZ-X810 microscope during image acquisition.**

| **Microscopy channel** | **Brightfield** | **GFP** | **TRITC** | **DAPI** |
| --- | --- | --- | --- | --- |
| Filter | None | OP-87763 BZX filter GFP | OP-87764 BZX filter TRITC | OP-87762 BZX filter DAPI |
| Excitation wavelength [nm] | - | 470/40 | 545/25 | 360/40 |
| Adsorption wavelength [nm] | - | 525/50 | 605/70 | 460/50 |
| Dichroic mirror wavelength [nm] | - | 495 | 565 | 400 |
| Exposure time [s] | 1/7500 | 7 | 1/3 | 1/100 |

**Table S3: DSM numbers and NCBI accession numbers of cultivated *Streptomyces* species.** Solid line indicates different morphological types.

| Morphology | Species | DSM Number | NCBI accession number |
| --- | --- | --- | --- |
| Pellet-forming | ***S. rimosus*** | 40260 | ASM33118v2 |
|  |  |  |  |
|  | ***S. fradiae*** | 40063 | ASM870442v1 |
|  |  |  |  |
|  | ***S. filamentosus*** | 40022 | ASM863401v1 |
|  |  |  |  |
|  | ***S. coeruleorubidus*** | 40145 | ASM870513v1 |
|  |  |  |  |
|  | ***S. avermitilis*** | 46492 | GCF_000009765.2 |
|  |  |  |  |
|  | ***S. alboniger*** | 40043 | ASM870439v1 |
|  |  |  |  |
|  | ***S. bobili/ S. galilaeus*** | ATCC 14969 | ASM870457v1 |
|  |  |  |  |
|  | ***S. cinereoruber*** | 40012 | ASM929938v1 |
|  |  |  |  |
|  | ***S. albofaciens*** | 40268 | ASM863402v1 |
|  |  |  |  |
| Dispersed | ***S. collinus*** | 40733 | ASM44487v1 |
|  |  |  |  |
|  | ***S. venezuelae*** | 40230 | ASM863916v1 |
|  |  |  |  |
|  | ***S. nodosus*** | 40109 | ASM1420503v1 |
|  |  |  |  |
|  | ***S. nitrosporeus*** | 40023 | ASM870455v1 |
|  |  |  |  |

1.4 **Figures**

**Figure S1: Results of LC-MS/MS quality control metrics.** (Median MS1 error in PPM, median retention time shift, total number of MS2 spectra per sample, median log2 intensity per sample, and number of detected features).

# 2 Supplemental Results

2.1 **Tables**

**Table S4: Comparison of growth rates derived from autofluorescence and scattered-light signal, for cultivations without cellulose.** For 10 of the 13 species, the initial growth rate, based on the autofluorescence and scattered-light signals, is not significantly different (paired t-test, α=0.05) during cultivation without cellulose addition.

| Morphology | Species | Time range [h] | Signal | Mean μ_1_ | p value |
| --- | --- | --- | --- | --- | --- |
| Pellet-forming | ***S. rimosus*** | 5.19 – 15.03 | autofluorescence | 0.214 ± 0.004 | 0.182 |
|  |  |  | scattered-light | 0.208 ± 0.008 |  |
|  | ***S. fradiae*** | 7.44 – 14.19 | autofluorescence | 0.277 ± 0.008 | 0.934 |
|  |  |  | scattered-light | 0.279 ± 0.019 |  |
|  | ***S. filamentosus*** | 2.44 – 13.44 | autofluorescence | 0.280 ± 0.014 | 0.242 |
|  |  |  | scattered-light | 0.256 ± 0.008 |  |
|  | ***S. coeruleorubidus*** | 3.44 – 28.03 | autofluorescence | 0.097 ± 0.004 | **0.004** |
|  |  |  | scattered-light | 0.122 ± 0.002 |  |
|  | ***S. avermitilis*** | 5.28 – 17.36 | autofluorescence | 0.113 ± 0.003 | 0.276 |
|  |  |  | scattered-light | 0.103 ± 0.006 |  |
|  | ***S. alboniger*** | 6.11 – 18.11 | autofluorescence | 0.166 ± 0.002 | **0.002** |
|  |  |  | scattered-light | 0.207 ± 0.004 |  |
|  | ***S. bobili*** | 3.53 – 22.45 | autofluorescence | 0.142 ± 0.009 | 0.772 |
|  |  |  | scattered-light | 0.138 ± 0.022 |  |
|  | ***S. cinereoruber*** | 6.19 – 10.69 | autofluorescence | 0.209 ± 0.052 | 0.129 |
|  |  |  | scattered-light | 0.122 ± 0.003 |  |
|  | ***S. albofaciens*** | 6.78 – 14.53 | autofluorescence | 0.329 ± 0.002 | 0.085 |
|  |  |  | scattered-light | 0.309 ± 0.009 |  |
| Dispersed | ***S. collinus*** | 4.36 – 13.78 | autofluorescence | 0.152 ± 0.004 | 0.382 |
|  |  |  | scattered-light | 0.147 ± 0.007 |  |
|  | ***S. venezuelae*** | 1.78 – 12.11 | autofluorescence | 0.235 ± 0.013 | 0.618 |
|  |  |  | scattered-light | 0.245 ± 0.014 |  |
|  | ***S. nodosus*** | 11.28 – 38.78 | autofluorescence | 0.060 ± 0.001 | **0.006** |
|  |  |  | scattered-light | 0.085 ± 0.002 |  |
|  | ***S. nitrosporeus*** | 2.78 – 44.28 | autofluorescence | 0.070 ± 0.001 | 0.203 |
|  |  |  | scattered-light | 0.077 ± 0.004 |  |

**Table S5: Detailed description of natural products and their annotation by SIRIUS.**

| **Name** | **m/z** | **Retention time [min]** | **Predicted molecular formula** | **Confidence/COSMIC score** | **Sirius formula score** | **Tanimoto similarity [%]** | **Species** | **Reference** |
| --- | --- | --- | --- | --- | --- | --- | --- | --- |
| Amphotericin B | 924.496 | 10.16 | C_47_H_73_NO_17_ | 0.370 | 36.06 | 91.701 | ***S. nodosus*** | (Donovick et al. 1955) |
| Mocimycin | 797.422 | 11.40 | C_43_H_60_N_2_O_12_ | 0.686 | 93.6 | 90.833 | ***S. collinus*** | (Wolf, Zahner, and Nierhaus 1972) |
| Oligomycin | 813.512 | 16.56 | C_45_H_74_O_11_ | 0.640 | 99.87 | 92.308 | ***S. avermitilis*** | (Lin et al. 2009) |
| Staurosporin | 467.208 | 9.10 | C_28_H_26_N_4_O_3_ | 0.983 | 75.1 | 97.234 | ***S. fradiae*** | (Guan et al. 2019) |
| Daunomycin | 528.187 | 9.07 | C_27_H_29_NO_10_ | 0.851 | 99.1 | 97.083 | ***S. coeruleorubidus*** | (Blumauerová et al. 1977) |
| Rimocidin | 768.417 | 9.96 | C_39_H_61_NO_14_ | 0.511 | 99.95 | 69.804 | ***S. albofaciens*** |  |
|  |  |  |  |  |  |  | ***S. rimosus*** | (Davisson et al. 1951) |
| Tetracycline | 445.162 | 6.35 | C_22_H_24_N_2_O_8_ | 0.937 | 99.98 | 95.714 | ***S. rimosus*** |  |
|  |  |  |  |  |  |  | ***S. albofaciens*** |  |
| Aklavine/ Aclacinomycin T | 570.233 | 9.88 | C_30_H_35_NO_10_ | 0.917 | 99.94 | 82.061 | ***S. bobili*** |  |
| Aclacur/ Aclacinomycin A | 812.348 | 10.92 | C_42_H_53_NO_15_ | 0.560 | 74.9 | 85.714 | ***S. bobili*** | (Oki et al. 1980) |

**Table S6: Additional Mantel tests confirming the robustness of BGC and metabolome correlation.**

| **Test** | **Subset of metabolites filtered by CANOPUS annotation** | **Spearman** ρ | **p-value** |
| --- | --- | --- | --- |
| Mantel test | full metabolome | 0.578 | 0.0005 |
| Mantel test | polyketides/macrolides and other specialized metabolites | 0.549 | 0.0006 |
| Mantel test | polyketides/macrolides, other specialized metabolites, benzenoids, and specialized scaffolds | 0.57 | 0.0016 |
| Partial Mantel test | metabolome, controlled for phylogenetic distance | 0.568 | 0.0007 |

5.1 **Figures**

**Figure S2: Growth curves of scattered-light (SL) without cellulose (grey), autofluorescence (AF) without cellulose (green), and with cellulose (blue) for dispersed *Streptomyces* spp. (n = 3 per species and condition).** Dashed purple line indicates presumed secondary substrate limitation, dotted black line indicates presumed glucose limitation. A: *S. venezuelae*, B: *S. collinus*, C: *S. nodosus*, D: *S. nitrosporeus.*

**Figure S3: Growth curves of autofluorescence (AF) without cellulose (green) and with cellulose (blue), scattered-light (SL) without cellulose (dark grey) for pelleted *Streptomyces* spp. (n = 3 per species and condition).** Dashed purple line indicates presumed secondary substrate limitation, dotted black line indicates presumed glucose limitation. A: *S. coeruleorubidus*, B: *S. cinereoruber*, C: *S. filamentosus*, D: *S. avermitilis*, E: *S. bobili*, F: *S. fradiae,* where the scattered-light measurement (light brown) with cellulose shows a drop, which could potentially be a dispersal event of the pellets. G: *S. albofaciens*, H: *S. rimosus*, I: *S. alboniger*.

**Figure S4: Phylogenetic tree of *Streptomyces* spp. created with autoMLST.** The outgroup was *Micromonospora inositola*.

**Figure S5: Response tanglegram comparing the biosynthetic potential vs. the metabolic response (+ cellulose minus - cellulose).** The increase in entanglement (0.10 vs. 0.04 at glucose baseline) visually demonstrates a weaker correspondence between biosynthetic potential and the cellulose-associated response than in the glucose-only metabolome.

**Figure S6: Evaluation of cellulase activity showed no relevant activity in *S. albofaciens, S. avermitilis, S. bobili,* and *S. coeruleorubidus.* A:** After 6 days of shake flask cultivation (10 ml, 200 rpm, 50 mm shaking diameter) in LNP medium containing 30 gL^-1^ glucose and 30 gL^-1^ cellulose, none of the *Streptomyces* cultivations exceeded the theoretical OTR integral limit (1000 mmolL^-1^), corresponding to complete respiration of supplied glucose. Comparatively, *Trichoderma reesei* RUT-C30, which was grown on 5 gL^-1^ glucose and 30 gL^-1^ cellulose, substantially exceeded the theoretical OTR integral limit (166.5 mmolL^-1^). **B:** Filter paper assays showed that, in comparison to supernatant from positive control (*Trichoderma reesei* RUT-C30*)*, supernatants from *Streptomyces* species did not cause glucose release from the cellulose filter paper after 1 h of incubation at 50°C.

# 6 References

Blumauerová, M., J. Matějů, K. Stajner, and Z. Vaněk. 1977. "Studies on the production of daunomycinone derived glycosides and related metabolites in *Streptomyces coeruleorubidus* and *Streptomyces peucetius*." *Folia Microbiologica* 22 (4): 275-285. <https://doi.org/10.1007/BF02877657>. <https://doi.org/10.1007/BF02877657>.

Davisson, J. W., F. W. Tanner, Jr., A. C. Finlay, and I. A. Solomons. 1951. "Rimocidin, a new antibiotic." *Antibiot Chemother (Northfield)* 1 (5): 289-90.

Donovick, R., W. Gold, J. F. Pagano, and H. A. Stout. 1955. "Amphotericins A and B, antifungal antibiotics produced by a streptomycete. I. In vitro studies." *Antibiot Annu* 3: 579-86.

Guan, Hanye, Yue Li, Jiazhen Zheng, Ning Liu, Jihui Zhang, and Huarong Tan. 2019. "Important role of a LAL regulator StaR in the staurosporine biosynthesis and high-production of *Streptomyces fradiae* CGMCC 4.576." *Science China Life Sciences* 62 (12): 1638-1654. <https://doi.org/10.1007/s11427-019-1597-6>. <https://doi.org/10.1007/s11427-019-1597-6>.

Lin, X., Y. Wen, M. Li, Z. Chen, J. Guo, Y. Song, and J. Li. 2009. "A new strain of *Streptomyces avermitilis* produces high yield of oligomycin A with potent anti-tumor activity on human cancer cell lines in vitro." *Appl Microbiol Biotechnol* 81 (5): 839-45. <https://doi.org/10.1007/s00253-008-1684-y>. <https://www.ncbi.nlm.nih.gov/pubmed/18810431>.

Oki, T., A. Yoshimoto, Y. Matsuzawa, T. Takeuchi, and H. Umezawa. 1980. "Biosynthesis of anthracycline antibiotics by *Streptomyces galilaeus*. I. Glycosidation of various anthracyclinones by an aclacinomycin-negative mutant and biosynthesis of aclacinomycins from aklavinone." *J Antibiot (Tokyo)* 33 (11): 1331-40. <https://doi.org/10.7164/antibiotics.33.1331>.

Prauser, H., and R. Falta. 1968. "Phagensensibilität, Zellwand-Zusammensetzung und Taxonomie von Actiomyceten." *Z Allg Mikrobiol* 8: 39-46.

Wolf, H., H. Zahner, and K. Nierhaus. 1972. "Kirromycin, an inhibitor of the 30 S ribosomal subunits function." *FEBS Lett* 21 (3): 347-350. <https://doi.org/10.1016/0014-5793(72)80199-6>. <https://www.ncbi.nlm.nih.gov/pubmed/11946543>.
